# FDG PET in the differential diagnosis of degenerative parkinsonian disorders: usefulness of voxel-based analysis in clinical practice

**DOI:** 10.1007/s10072-022-06166-w

**Published:** 2022-06-14

**Authors:** Annachiara Arnone, Michela Allocca, Rossella Di Dato, Giulia Puccini, Iashar Laghai, Federica Rubino, Matilde Nerattini, Silvia Ramat, Gemma Lombardi, Camilla Ferrari, Valentina Bessi, Sandro Sorbi, Maria Teresa De Cristofaro, Cristina Polito, Valentina Berti

**Affiliations:** 1 Nuclear Medicine Unit, Department of Experimental and Clinical Biomedical Sciences “Mario Serio”, University of Florence, 50134, Florence, Italy; 2 Department of Nuclear Medicine, Hospital of Prato, Via Suor Niccolina Infermiera, 20/22, 59100, Prato, Italy; 3 Parkinson Unit, Department of NeuroMuscular- Skeletal and Sensorial Organs, AOU Careggi, Florence, Italy; 4 Department of Neuroscience, Psychology, Drug Research and Child Health (NEUROFARBA), University of Florence, Florence, Italy

**Keywords:** Degenerative Parkinsonian disorders, SPM, Voxel-based analysis, Positron emission tomography, Metabolism maps, Differential diagnosis

## Abstract

**Background:**

The early differential diagnosis among neurodegenerative parkinsonian disorders becomes essential to set up the correct clinical-therapeutic approach. The increased utilization of [^18^F] fluoro-deoxy-glucose positron emission tomography (FDG PET) and the pressure for cost-effectiveness request a systematic evaluation and a validation of its utility in clinical practice. This retrospective study aims to consider the contribution, in terms of increasing accuracy and increasing diagnostic confidence, of voxel-based FDG PET analyses in the differential diagnosis of these disorders, including Parkinson’s disease, multiple system atrophy, progressive supranuclear palsy, and cortico-basal syndrome.

**Method:**

Eighty-three subjects with a clinically confirmed diagnosis of degenerative parkinsonian disorders who underwent FDG brain PET/CT were selected. A voxel-based analysis was set up using statistical parametric mapping (SPM) on MATLAB to produce maps of brain hypometabolism and relative hypermetabolism. Four nuclear physicians (two expert and two not expert), blinded to the patients’ symptoms, other physicians’ evaluations, and final clinical diagnosis, independently evaluated all data by visual assessment and by adopting metabolic maps.

**Results:**

In not-expert evaluators, the support of both hypometabolism and hypermetabolism maps results in a significant increase in diagnostic accuracy as well as clinical confidence. In expert evaluators, the increase in accuracy and in diagnostic confidence is mainly supported by hypometabolism maps alone.

**Conclusions:**

In this study, we demonstrated the additional value of combining voxel-based analyses with qualitative assessment of brain PET images. Moreover, maps of relative hypermetabolism can also make their contribution in clinical practice, particularly for less experienced evaluators.

## Introduction

Atypical Parkinsonian disorders (APD) is a group of heterogeneous neurodegenerative diseases combining parkinsonian key features (bradykinesia, rigidity, and rest tremor) to dementia and to other clinical signs and symptoms related to the cortex or cerebellum [1]. Differently from Parkinson’s disease (PD), these disorders are characterized not only by cell loss in the substantia nigra, but also by degeneration in added nervous system areas that normally contain dopamine receptors, known as the striatum, and usually do not respond well to drug treatment as levodopa [2]. The more severe evolution and prognosis require different therapeutic approaches. Therefore, the early differential diagnosis among APD, often complicated by overlapping clinical presentations, becomes essential to set up the correct clinical-therapeutic approach [3]. Brain imaging with positron emission tomography (PET) and [^18^F]fluoro-deoxy-glucose (FDG) has been widely used in clinical practice as an important tool for revealing in vivo changes at different stages [4] and could represent a valid aid for the differential diagnosis of APD [5, 6]. In literature, many studies have described and considered frequent disease-related metabolic brain patterns. In PD, functional imaging has shown heterogeneous FDG PET patterns of hypometabolism, ranging from no cortical hypometabolism to diffuse parieto-temporo-occipital hypometabolism, which has been often associated with relative hypermetabolism of the striatal nuclei, globus pallidus, midbrain, thalamus, and cerebellum, as well as at the primary sensory-motor cortex [7, 8]. However, the origin of such pattern would be consistent across studies using global brain activity as a reference for activity scaling [9]. In Lewy Body Dementia (LBD), subcortical structures are relatively spared, as in PD. However, the reduction of cortical metabolic activity is widespread and more marked in occipital and temporo-parietal cortices, while a less relevant involvement is demonstrated in parietal and frontal cortices [10, 11]. On the other hand, in patients with progressive supranuclear palsy (PSP), a widespread cortical hypometabolism has been discovered, in particular in the frontal cortex, mainly in medial and premotor areas, as well as in subcortical structures, like the basal ganglia, especially caudate nucleus, and the midbrain [8, 12]. Concerning to multiple system atrophy (MSA), studies with FDG PET revealed significant hypometabolism in the striatum and in particular in the putamen, mainly in the parkinsonian form, as well as in the midbrain and cerebellum, prevalent in the cerebellar form [8, 12].

Notoriously, cortico-basal syndrome (CBS) is a very heterogeneous syndrome referring to gradual neurodegeneration in several cerebral cortical areas as well as deep structures, such as the basal ganglia. However, the underlying pathologies may be different, including CBD, AD, and others, which result in slightly different clinical phenotypes and in different FDG PET patterns [13]. In this context, FDG PET could also represent a valid tool to support the differential diagnosis within the possible etiologies underlying CBS. The typical pattern of hypometabolism of CBS due to CBD (CBS_CBD_) is characterized by an asymmetrical distribution, involving frontal, parietal, temporal cortical areas, basal ganglia and thalamus of the same hemisphere [8, 12, 14, 15].

With the evaluation of such metabolic patterns, FDG PET imaging has obtained an important role as supportive feature in the differential diagnosis of APD subtypes [16–19]. Moreover, some studies described the increase of diagnostic accuracy through the implementation of semiquantitative analysis, in support of qualitative evaluation [8, 20].

In the context of dementing neurodegenerative diseases, the European Association of Nuclear Medicine (EANM) and the European Academy of Neurology (EAN) have already recommended the use of semi-automated analyses as a valid helpful tool to improve the visual reading of FDG PET images [21].

However, both the increased utilization of FDG PET in APD disorders and the pressure for cost-effectiveness request a systematic evaluation and a validation of its utility in clinical practice. An optimized statistical parametric mapping (SPM) routine for FDG PET data analysis in single subjects has already been validated for the differential diagnosis of dementia [22]. Some authors have also evaluated its possible supportive role in a cohort of patients with APD, observing a significant impact on the clinical work-up and prognosis, particularly at early stages when the presentation is uncertain [1, 23]. However, to our knowledge, in literature, it has never been evaluated the impact of the use of FDG PET semi-automated analyses in a clinical setting, neither the use of both hypo- and hypermetabolism maps, as a way to detect both regions of decreased activity and of preserved neuronal activity, which could be especially useful in parkinsonian disorders. Therefore, the purpose of our study is to evaluate the contribution, in terms of increasing accuracy and increasing diagnostic confidence, of voxel-based FDG PET analyses in the differential diagnosis of neurodegenerative parkinsonian disorders, including PD, MSA, PSP, and CBS_CBD_. In particular, it will be evaluated the increasing accuracy and confidence of different nuclear medicine physicians in detecting the specific metabolic patterns after visual reading of FDG PET images, with the aid of statistical maps of hypometabolism and also of relative hypermetabolism.

## Materials and methods

### Subjects

Eighty-three subjects with a clinically confirmed diagnosis of neurodegenerative parkinsonian disorders (56 PD, 10 PSP, 9 MSA, 8 CBS_CBD_) who underwent FDG brain PET/CT in the period between January 2013 and December 2019 at the SOD of the Nuclear Medicine of AOU Careggi were selected. The confirmed clinical diagnosis has been made by neurologists after a period of at least 1 year, supported by FDG PET results (only qualitative assessment) but mainly based on symptoms evolution and response to treatments [24–31]. Due to the retrospective nature of the study, clinicians were not blinded with respect to FDG PET results.

### PET acquisition and processing

PET scans were executed 30–40 min after ^18^F-FDG administration (3.7 MBq/kg) according to EANM guidelines for brain imaging. Images were obtained on a PET/CT scanner (Philips Gemini TF 16 PET/CT) and reconstructions were performed using 3D LOR iterative algorithm reconstruction (FOV: 256, matrix: 128 × 128, voxel dimensions: 2 × 2 × 2 mm). CT acquisition for attenuation correction was performed on spiral 16 slices CT with a slice thickness of 2 mm.

### SPM analysis

A voxel-based analysis was set up using SPM12 on MATLAB (MathWorks Inc., Sherborn, MA, USA). Images were anonymized, manually reoriented, setting the origin to the anterior commissure, normalized according to the FDG template, and then smoothed (FWHM 8 mm) [32]. The control group has been provided by Neurological Study Group AIMN [33]. Comparisons between each patient and controls have been performed using independent samples *T*-test routine, with age as a nuisance variable and activity proportional scaling to the global mean value. The significance threshold was set at *p* < 0.001, uncorrected for multiple comparisons. Only clusters containing more than 16 voxels were deemed to be significant. T-maps of both relative hypo- and hypermetabolism were visualized on the standardized T1-MRI provided by SPM. The performance of FDG PET qualitative assessment and SPM t-maps classification has been evaluated using multinomial logistic regression analysis. Cohen’s *k* coefficient was run to determine the agreement between readers.

### Image evaluation

All images were independently evaluated by 4 nuclear physicians (2 experts and 2 not expert), blinded to the patients’ symptoms, other physicians’ evaluations, and final clinical diagnosis. Expert evaluators were defined as nuclear physicians with at least 5 years of experience in evaluating and reporting brain FDG PET examinations. Not-expert evaluators were physicians in specialty training in Nuclear Medicine, who were familiar with the type of examination, but with little experience in their work practice.

Each reader had to assign to each patient a specific degenerative parkinsonism pattern (among PD, MSA, PSP, CBS_CBD_) and a confidence level (on a scale of 0 to 100).

**Fig. 1:**
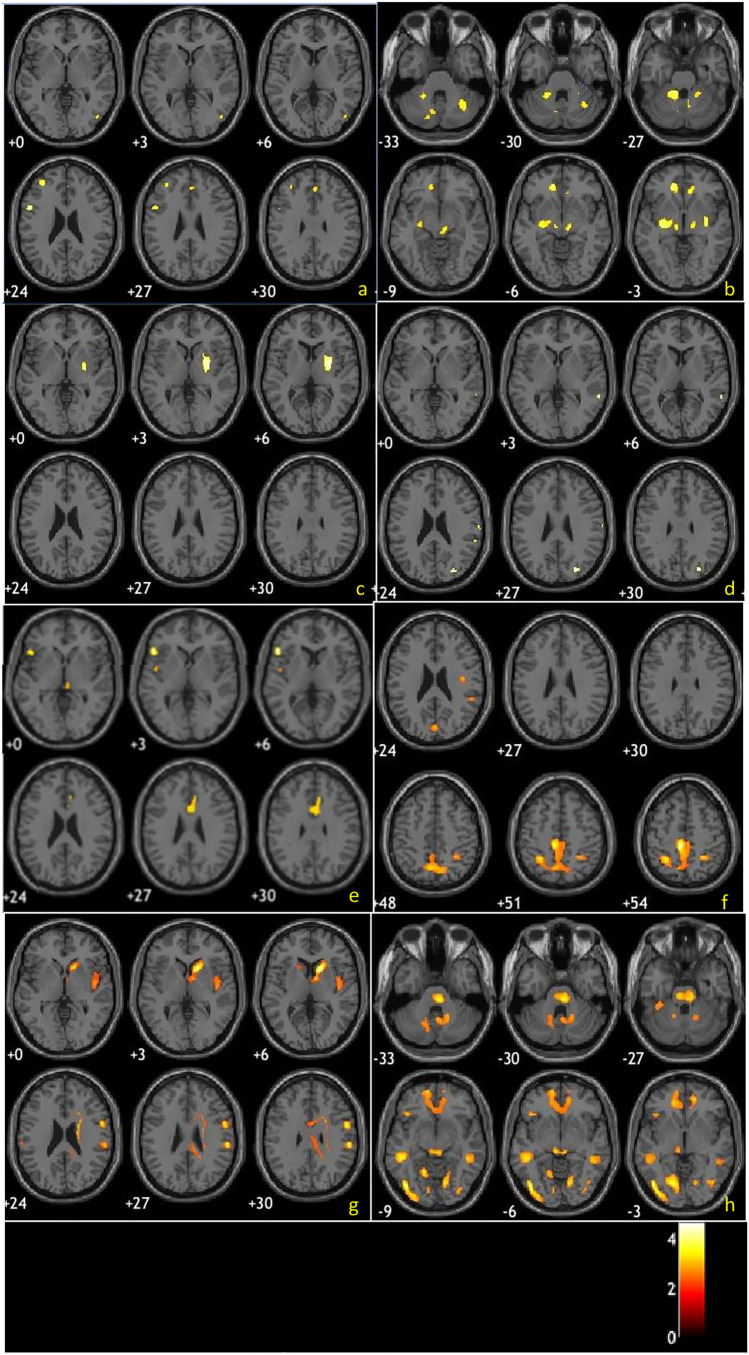
Hypo- and hypermetabolic T-maps of four patients, one for each disease in the differential diagnosis. PD (**a**–**b**), MSA (**c**–**d**), PSP (**e**–**f**), and CBS_CBD_ (**g**–**h**), respectively

Firstly, FDG PET qualitative assessment on a general color scale, usually adopted in clinical practice, was performed [12]. Then, the evaluators had to confirm or modify the diagnosis, indicating again the confidence level, based on the visualization of the hypometabolism maps. Finally, maps of relative hypermetabolism were also used to complete the whole evaluation. Hypo- and hypermetabolic T-maps of four patients are represented in Fig. 1.

### Statistical analysis

Statistical analyses were performed using IBM® SPSS® Statistics, setting the significance threshold at *p* < 0.01.

## Results

### Expert readers

The expert readers correctly classified 85.54% of the patients after qualitative assessment and 91.57% of the patients after the evaluation of the hypometabolism and hypermetabolism maps. The percentages of patients correctly classified after each assessment are shown in Table 1.

**Table 1:** Accuracy values obtained by expert readers’ evaluation

	Qualitative assessment	Additional hypometabolism maps assessment	Additional hypermetabolism maps assessment
PD	92.86	96.43	96.43
MSA	77.78	100	100
PSP	80	90	90
CBS	50	50	50
%cc total	85.54	91.57	91.57

The percentage of patients correctly classified did not change for CBS, while significantly increased from 77.78 to 100% for MSA, from 92.86 to 96.43% for PD, and from 80 to 90% for PSP. In all cases, the percentage of correctly classified subjects did not increase after the evaluation of hypermetabolism maps. Diagnostic confidence values in general and by individual disease were reported in Table 2. Confidence in diagnosis after qualitative assessment and after evaluation of voxel-based maps is significantly increasing, overall and for each individual disease group. In general, diagnostic confidence increases from 80.6% by qualitative assessment to 87.11% with the help of hypometabolism maps and to 90.84% after assessment also of relative hypermetabolism maps. The values rise from 81.88 to 92.32% for PD; there is an increase from 81.11 to 90.56% for MSA; from 72.5 to 84.5% for PSP; finally, the increase is from 81.25 to 88.75% for CBS.

**Table 2:** Diagnostic confidence values obtained by expert readers’ evaluation

Diagnostic confidence (0–100)	Qualitative evaluation	Additional hypometabolism maps assessment	Additional hypermetabolism maps assessment
Mean	80.60	87.11	90.84
Mean PD	81.88	87.68	92.32
Mean MSA	81.11	88.89	90.56
Mean PSP	72.50	81.50	84.50
Mean CBS	81.25	88.13	88.75

For Expert readers, sensitivity/specificity/PPV/NPV of a PD conclusion for PD diagnosis at qualitative assessment were respectively: 92.9%, 74.1%, 88.1%, and 88.1%; for MSA, 77.8%, 97.3%, 77.8%, and 77.8%, respectively; for PSP, 80%, 97.3%, 80%, and 80%, respectively; and for CBS, 50%, 98. 7%, 80%, and 80%, respectively.

Sensitivity/specificity/PPV/NPV of a PD conclusion for PD diagnosis at hypo maps assessment were respectively: 96.4%, 85.2%, 93.1%, and 93.1%; for MSA, 100%, 97.3%, 81.8%, and 81.8%, respectively; for PSP, 90%, 100%, 100%, and 100%, respectively; and for CBS, 50%, 98.7%, 80%, and 80%, respectively. 

Sensitivity/specificity/PPV/NPV of a PD conclusion for PD diagnosis at hyper maps assessment were respectively 96.4%, 85.2%, 93.1%, and 93.1%; for MSA, 100%, 97.3%, 81.8%, and 81.8%, respectively; for PSP, 90%, 100%, 100%, and 100%, respectively; and for CBS, 50%, 98.7%, 80%, and 80%, respectively.

### Not-expert readers

Not-expert readers correctly classified 61.45% of patients after qualitative assessment, 74.7% after the help of the hypometabolism maps, and 81.93% after also assessing the relative hypermetabolism maps. Accuracy values for individual pathology and overall accuracy results are shown in Table 3. The diagnostic accuracy did not change for CBS. It increased from 66.67 to 88.89% for MSA. The percentage of correctly classified subjects increased progressively from 62.50 to 85.71% for PD and from 60 to 80% for PSP.

**Table 3:** Accuracy values obtained by not-expert readers’ evaluation

	Qualitative assessment	Additional hypometabolism maps assessment	Additional hypermetabolism maps assessment
PD	62.50	76.79	85.71
MSA	66.67	88.89	88.89
PSP	60	70	80
CBS	50	50	50
%cc total	61.45	74.70	81.93

The overall and single pathology diagnostic confidence values were reported in Table 4. Confidence in correct diagnosis after qualitative assessment and after evaluation of hypo- and hypermetabolism maps shows a statistically significant increase, both overall and by single pathology (*p* < 0.01). Such classification resulted in statistically significant in predicting follow-up clinical diagnosis at multinomial logistic regression analysis (*p* < 0.001).

**Table 4:** Diagnostic confidence values obtained by not expert readers’ evaluation

Diagnostic confidence (0–100)	Qualitative evaluation	Additional hypometabolism maps assessment	Additional hypermetabolism maps assessment
Mean	77.71	83.73	88.31
Mean PD	78.04	82.68	88.57
Mean MSA	85.56	94.44	95.56
Mean PSP	69	79	80
Mean CBS	77.50	85	88.75

For non-expert readers, sensitivity/specificity/PPV/NPV of a PD conclusion for PD diagnosis at qualitative assessment was respectively: 62.5%, 77.8%, 85.4%, and 85.4%; for MSA, 66.7%, 86.5%, 37.5%, and 37.5%, respectively; for PSP, 60%, 89%, 42.9%, and 42.9%, respectively; and for CBS, 50%, 89.3%, 33.3%, and 33.3%, respectively.

Sensitivity/specificity/PPV/NPV of a PD conclusion for PD diagnosis at hypo maps assessment was respectively 76.8%, 81.2%, 89.6%, and 89.6%, respectively; for MSA, 88.9%, 89.2%, 50%, and 50%, respectively; for PSP, 70%, 91.8%, 53.8%, and 53.8%, respectively; and for CBS, 50%, 97.3%, 66.7%, and 66.7%, respectively.

Sensitivity/specificity/PPV/NPV of a PD conclusion for PD diagnosis at hyper maps assessment was respectively 85.7%, 85.2%, 92.3%, and 92.3%; for MSA, 88.9%, 90.5%, 53.3%, and 53.3%, respectively; for PSP, 80%, 97.3%, 80%, and 80%, respectively; and for CBS, 50%, 97.3%, 66.7%, and 66.7%, respectively.

**Fig. 2:**
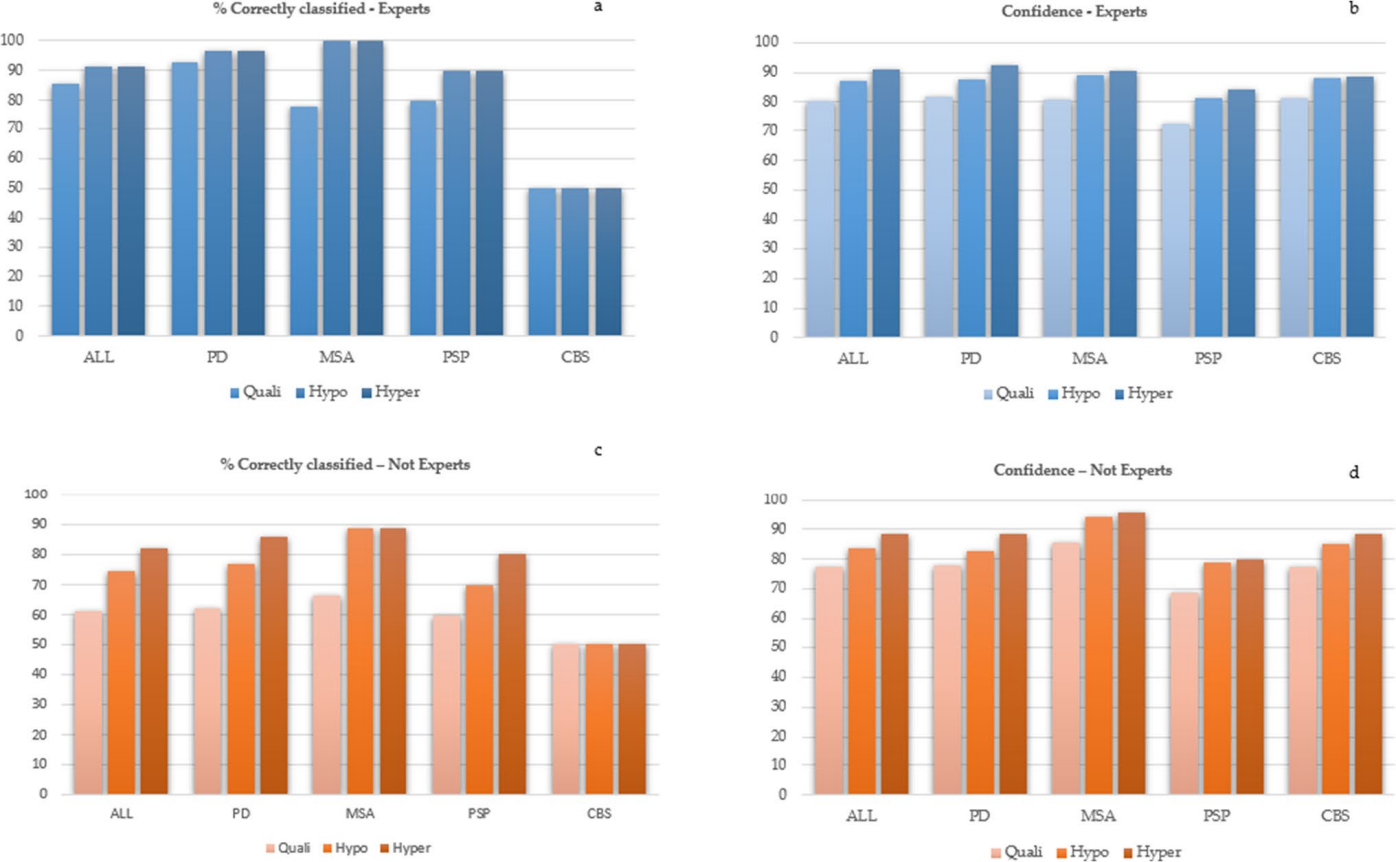
Graphics summarizing the estimation in terms of percentage of correctly classified patients and the confidence level respectively by experts (**a**–**b**) and not experts (**c**–**d**). Colors were set in order to highlight the added value of hypometabolism and hypermetabolism maps compared to the qualitative assessment alone, as indicated in the legend

Results have been graphically summarized in Fig. 2. Between both expert and non-expert readers, Cohen’s *k* coefficent stated agreement regarding qualitative, hypometabolic, and hypermetabolic maps (*k* = 0.397, *k* = 0,627, *k* = 0,746, respectively. *p* < 0.001) (Fig. 2).

## Discussion

Our study takes part in the well-known debate about the utility of metabolic biomarkers in the diagnostic work-up of APD, in order to obtain benefits for treatment options and support to the patients [1, 23]. In particular, we aim to underline the clinical practice role of FDG PET/CT imaging in the differential diagnosis among neurodegenerative parkinsonian disorders. Our specific focus was to evaluate the additional value, over qualitative PET images assessment, of not only maps of hypometabolism, as a mean to evaluate regions of neuronal dysfunction and loss, but also maps of relative hypermetabolism, as a way to evaluate cerebral regions of preserved synaptic activity.

Several studies in the literature have demonstrated the significant value of voxel-based analyses as an aid in brain FDG PET assessment in patients with cognitive decline [16–19, 21, 22]. Similar results are also expected in the field of APD, although there are no studies that have ever formalized this result. First of all, the results of our study show that the diagnostic accuracy values are higher in expert evaluators than in not-experts, especially regarding the qualitative assessment of PET images. This result was obviously expected and it emphasizes the complexity of brain FDG PET evaluation, which requires considerable work experience. For expert evaluators, overall high accuracy just after qualitative assessment increases with the use of hypometabolism maps and concurrent increase also in confidence levels and reaches stability after the use of hypermetabolism maps. It suggests that they could not be completely useless in this group of evaluators. For not-expert readers, overall accuracy after qualitative assessment is suboptimal and the use of both hypometabolism and hypermetabolism maps let them arrive at levels of accuracy similar to expert readers, consequently increasing their levels of confidence. Regarding the different diseases considered in this study, we could express the following considerations.

In the cases of PD disease, experts reach the highest accuracy, suggesting that this pattern is well recognizable even if very heterogeneous actually including both forms of PD patients, either without cognitive deficits or with mild cognitive impairment, ranging from no cortical hypometabolism to some areas of posterior hypometabolism. Accuracy levels still improve after the use of hypometabolism maps but do not change after the use of hypermetabolism maps, suggesting that the typical relative hypermetabolism in striata is well recognizable by the experts. Instead, not experts’ accuracy is quite low after qualitative assessment, suggesting that the widespread hypometabolism is difficult to recognize by them. However, with the use of both hypometabolism and hypermetabolism maps, accuracy increases of around 10% each time, reaching good levels, suggesting that voxel-based assessment is fundamental for not-expert readers. However, they did not reach the levels of accuracy of experts. Regarding confidence, it is quite high and in both groups increases at each evaluation step. In the cases of MSA disease, accuracy after the qualitative assessment is one of the lowest for the experts; however, it reaches 100% after the use of hypometabolism maps. This suggests that hypometabolism maps are really relevant even in experts’ readers to identify the MSA patterns. In not experts, it happens almost the same thing. After hypometabolism maps, they reached the highest accuracy among diseases, and it did not improve after hypermetabolism maps. In both groups, however, the use of hypermetabolism maps increases confidence, maybe because of the confirmation that there is a metabolic sparing of cortical regions [24, 25]. In the cases of PSP disease, one of the lowest accuracy levels after qualitative assessment happens in both experts and not experts, improving after hypometabolism maps in both groups and after hypermetabolism maps only in not experts. This could suggest the difficulty in evaluating this pathology, which could present with different patterns in the different variants [26]. This lower accuracy level as compared to the other pathologies is reflected by the confidence, which is the lowest among pathologies. In the cases of CBS disease, diagnostic accuracy values are rather low in both expert and not-expert readers. This is in line with the literature since the typical CBS-specific metabolic pattern is actually present only in the subgroup of patients with cortico-basal degeneration (CBD) pathology, while others could show metabolic patterns mimicking other diseases [13, 27]. It also seems that when the specific pattern is present (cortical and subcortical hypometabolism with frank asymmetry), it is well recognizable even only with qualitative assessment, and even by not-expert evaluators, since the percentages of correctly classified subjects did not improve after the use of any statistical maps. For expert evaluators, it can be noted an increase in accuracy only with the help of the hypometabolism maps, while it remains at the same level after the evaluation of the relative hypermetabolism maps. This result suggests that expert readers probably are able to evaluate which regions are spared by disease already from the qualitative assessment, and do not need further analysis. Moreover, the accuracy and the confidence values increase significantly with the help of the hypometabolism maps, so we derived that experts find valuable help in this type of analysis. On the other hand, even if relative hypermetabolism maps did not increase accuracy values for experts, diagnostic confidence seems to grow up. It can be deduced that this type of analysis is not completely useless, but contributes to increase the certainty with which the diagnosis is proposed. With regard to not-expert evaluators, the use of both hypo- and hypermetabolism maps proves to be very useful. In fact these evaluators, starting from rather low levels of accuracy at qualitative assessment alone, reaches much better levels, not equal to those of the experts but however very close to them and clinically acceptable. It is inferred that the not-expert evaluators need to make use of further analysis to better understand where both the disease-affected and disease-spared regions are located. It is also interesting to notice that the diagnostic confidence in such evaluations increases significantly as accuracy increases.

## Conclusions

Early diagnosis of neurodegenerative parkinsonian disorders is a crucial point of interest because both prognosis in terms of disease duration and clinical progression, and treatment options can vary considerably among the various subtypes. In this study, we demonstrated the additional value of combining voxel-based analyses with a qualitative assessment of brain PET images. It is shown that metabolic maps are very useful in supporting this type of evaluation, especially for less experienced nuclear physicians, but also for experienced ones. In not-expert evaluators, the support results in a significant increase in diagnostic accuracy as well as clinical confidence. In expert evaluators, the increase in accuracy is supported by hypometabolism maps alone, and there is still an increase in diagnostic confidence. Finally, in this particular group of diseases, it was shown that even maps of relative hypermetabolism can make their contribution to clinical practice, particularly for less experienced evaluators.
